# Considering Global Development? Insights from Applications for FDA Breakthrough Therapy and EMA PRIME Designations

**DOI:** 10.1007/s43441-022-00475-0

**Published:** 2022-10-28

**Authors:** Zahra Hanaizi, Sandra Kweder, Shannon Thor, Sonia Ribeiro, Anabela Marcal

**Affiliations:** 1 Human Medicines Division, Scientific Evidence Generation Department, European Medicines Agency, Domenico Scarlattilaan 6, 1083 HS Amsterdam, The Netherlands; 2 Office of Global Policy and Strategy, US Food and Drug Administration, Silver Spring, MD USA; 3 Europe Office, US Food and Drug Administration, Silver Spring, MD USA; 4 International Affairs Department, European Medicines Agency, Amsterdam, The Netherlands

**Keywords:** Drug development, Regulatory, EMA, FDA, PRIME, Breakthrough

## Abstract

The United States Food and Drug Administration and the European Medicines Agency (EMA) each have programs to expedite development of products identified as having potential to address unmet medical needs: the Breakthrough therapy (BT) and Regenerative Medicines Advanced Therapies designation programs in the US and the Priority Medicines (PRIME) scheme at EMA. We reviewed commonalities and differences in requests submitted and products designated through these programs, with the intent to explore ways to better support global development. During the period from PRIME’s launch in April 2016 to 31 December 2020, 151 requests were made to both BT and PRIME programs and the agencies reached concordant outcomes to grant or deny requests for almost two thirds of the cases (93/151, 62%), suggesting similar perspectives across international regulators on the potential of the products under study. Forty-two (42/151, 28%) products were granted both BT and PRIME, thus found by both Agencies to have the potential to address an unmet need for a serious condition, and thereby products for which efficient development would be highly desirable. Working toward better engagement on global development strategies is in the best interests of patients and public health. With this in mind, Agencies and sponsors should take advantage of existing collaborative opportunities, such as parallel scientific advice, and work to identify fresh approaches to support global development of products for unmet medical needs.

## Introduction

The United States Food and Drug Administration (FDA) and the European Medicines Agency (EMA) each have programs to expedite development of products identified as having potential to address unmet medical needs. FDA’s Center for Drug Evaluation and Research (CDER) and Center for Biologics Evaluation and Research (CBER) Breakthrough therapy (BT) designation program (launched in 2012), and EMA’s Priority Medicines (PRIME) program (launched in 2016) are such programs that seek to provide support and scientific advice during product development. As unmet medical needs are often global, companies developing treatments to address them are often inclined to seek global markets. Therefore, when EMA initiated the PRIME scheme in 2016, the Agencies began to work together to monitor commonalities and differences in what was submitted and designated, with the intent to explore ways to better support global development if the data suggested that there were common designation outcomes in common timeframes. Herein we review the results of this collaboration with an eye toward opportunities for our agencies to continue to work together in advising companies for products designated both BT and PRIME.

## Background

FDA’s BT designation launches a process designed to expedite the development and review of drugs and biological products (hereafter ‘drugs’) intended to treat serious conditions and for which preliminary clinical evidence indicates that the drug may demonstrate substantial improvement over available therapies on a clinically significant endpoint(s). Whether the improvement over available therapies is substantial is a matter of judgment and depends on both magnitude of the treatment effect, which could include duration of the effect, and the importance of the observed clinical outcome. In general, the preliminary clinical evidence should show a clear advantage over available therapy. A drug that receives a BT designation is eligible for all FDA fast track designation features (expedited development, rolling reviews), intensive guidance on an efficient drug development program (beginning as early as Phase 1), and organizational commitment to involve senior managers in all aspects of the process [1, 2]. Key features of the FDA BT designation program, as well as EMA’s PRIME and FDA’s more recent CBER Regenerative Medicines Advanced Therapies (RMAT) designation programs are shown in Table 1.

**Table 1:** Comparison of breakthrough, PRIME, and RMAT designations

Designation	Breakthrough therapy [4]	Priority medicine [3]	Regenerative medicine advanced therapy [5]
Agency	FDA	EMA	FDA
Established	2012	2016	2018
Qualifying criteria	A drug that is intended to treat a serious condition, AND preliminary clinical evidence indicates that the drug may demonstrate substantial improvement on a clinically significant endpoint(s) over available therapies	A drug that is expected to offer a major therapeutic advantage over existing treatments, or benefit patients without treatment options, AND preliminary clinical data indicates that it has the potential to address an unmet medical need	A drug is a regenerative medicine therapy, AND the drug is intended to treat, modify, reverse, or cure a serious condition, AND preliminary clinical evidence indicates that the drug has the potential to address unmet medical needs for such disease or condition
Benefits	–All Fast Track designation features –Intensive guidance on efficient drug development –Organizational commitment involving senior managers	–Early and proactive support and scientific advice at key milestones –Early Rapporteur appointment to provide continuous support –Kick-off meeting with the CHMP/CAT rapporteur and a multidisciplinary group of experts; –Potential for accelerated assessment –Dedicated EMA point of contact for continuous support	–All breakthrough therapy designation features –Early interactions to discuss any potential surrogate or intermediate endpoints
Timeframe	Can be granted at any phase of development	Considers products in Phase 1 or 2	Can be granted at any phase of development
Authorized medicines eligible^a^	Yes, based on indication	No	Yes, based on indication
Requires substantial improvement over existing therapies	Yes	Yes	No

^a^Medicinal products already approved or licensed in their respective region

EMA’s PRIME scheme provides enhanced support for the development of medicines that target an unmet medical need. It, too, focuses on medicines that may offer a major therapeutic advantage over existing treatments, or benefit patients without treatment options. To be accepted for PRIME, data must show the product’s potential to benefit patients with unmet medical needs based on early clinical data. PRIME builds on EMA’s existing regulatory framework and tools already available such as scientific advice and accelerated assessment. A Rapporteur (one of the two members of the committee who will perform the scientific evaluation) and a dedicated EMA contact point are assigned early in the development for continuous support. A key feature of PRIME is the organization of a multi-disciplinary meeting (so called ‘kick-off meeting’) aimed at initiating the interaction between the sponsor,^1^ experts from the EU regulatory network and the Agency. This establishes a discussion platform for the tailored development support for PRIME products with a view to defining and planning technical and scientific assistance through scientific advice and/or other interactions with EU regulators. Developers can expect PRIME products to be eligible for accelerated assessment at the time of application for a marketing authorization [3].

Key differences between BT and PRIME requirements for designation are the stage of development at which a company’s product may be eligible, whether already authorized medicines may be considered and, to some extent, the evidentiary standard for inclusion.

- Stage of development: EMA’s PRIME focuses on medicines that are not yet authorized and still early in clinical development, whereas BT designation can be granted at any phase in the development (in practice, this may even be during phase 3 or just prior to submission of a marketing application) or for a new indication of an already authorized medicine. In rare cases, medicines already advanced in their development may also be included in PRIME if the support features of the scheme are still expected to benefit the development at that stage.
- Evidentiary: FDA’s BT requires preliminary clinical data that the drug may demonstrate substantial improvement on a clinically significant endpoint(s) over available therapies. While EMA’s PRIME is open to all companies on the basis of preliminary clinical evidence, sponsors from the academic sector and micro-, small-, and medium-sized enterprises (SMEs) can apply earlier on the basis of compelling non-clinical data and tolerability data from initial clinical trials.

In 2018, FDA launched an additional expedited program, the CBER RMAT program, similar in intent to CDER and CBER BT designation program, but focused on regenerative medicine therapies such as cell and certain gene therapies [5]. RMAT designation strives to facilitate development for regenerative medicine therapies intended to treat, modify, reverse, or cure a serious condition when, similar to BT, preliminary clinical evidence indicates that the drug has the potential to address unmet medical needs for such conditions. RMAT designation grants all the benefits of the fast track and BT designation programs, including early interactions with FDA. RMAT is also specifically intended to allow for discussion of potential surrogate or intermediate endpoints to support accelerated approval. An important difference between RMAT and BT is that the RMAT criteria do not require the drug to demonstrate substantial improvement over existing therapies or major therapeutic advantage.

In 2016, shortly after EMA’s launch of PRIME, EMA, and FDA scientists began to monitor and analyze respective designation outcomes for BT and PRIME. With the RMAT program’s launch in 2018, these designations were added to the data repository. Outcomes of development programs themselves, such as whether marketing applications were ultimately submitted for the subject products, were not part of this assessment.

## Methods

We began with a review of requests received for PRIME since its establishment in 2016 up to 31 December 2020. We next identified whether each of the products had been the subject of a request for BT designation for the same indication at FDA at any time (including before 2016) up to 31 December 2020. For any request received by both Agencies (referred to as common requests), we compared the designation outcomes and whether these were concordant (granted by both or denied by both Agencies) or divergent (granted by one and denied by the other Agency).^2^ We analyzed the distribution of common requests by therapeutic areas, including for concordance in designation. We also assessed whether requests common to both agencies were submitted within a similar timeframe (± 60 and ± 120 days) and whether this changed over the years.

Finally, while our primary interest is the products designated both as BT and PRIME, we thought it important to understand differences in FDA and EMA decisions on designation requests, defined as whether the request for designation was granted or denied. Therefore, we examined requests with divergent outcomes to identify the main reasons for divergence. This review included review documents from both agencies’ files and factored in whether the same or different data were submitted to justify the designation. If different data were submitted to FDA and EMA to support the applications, we did not assess the divergence further. If the data were the same, additional reasons were considered. Thus, the reasons were then classified under the following prespecified categories:

- Different data submitted in support of the application to the two agencies
- Differences of program implementation policy (e.g., a product too advanced in its development program may not be granted eligibility to PRIME by the EMA)
- Differences of currently available treatment across regions (thereby affecting assessment of whether the subject product may bring a major advantage/significant improvement over existing therapies)
- Differences in scientific judgement or interpretation of same data

In a secondary analysis for the same time period, we added RMAT to the assessment, comparing outcomes of requests submitted to PRIME and BT and/or RMAT. If a product had been subject to requests for both BT and RMAT with only one of these ‘granted’, the ‘granted’ designation was included in our comparison to PRIME.

## Results

### Comparison of Breakthrough and PRIME Submissions and Agency Outcomes

During the period 4 April 2016 to 31 December 2020, FDA received 700 BT designation requests while EMA had 348 requests for PRIME. In our comparison, we also considered 27 additional requests for BT designation submitted to FDA prior to April 2016, for which application to PRIME was requested for the same indication in the period covered by the analysis. One hundred and fifty-one requests were made to both programs. Thus, 43% (151/348) of the requests for EMA’s PRIME were also submitted to FDA for BT, and 21% (151/727) of the requests for FDA’s BT designation were also submitted to EMA for PRIME.

**Fig. 1:**
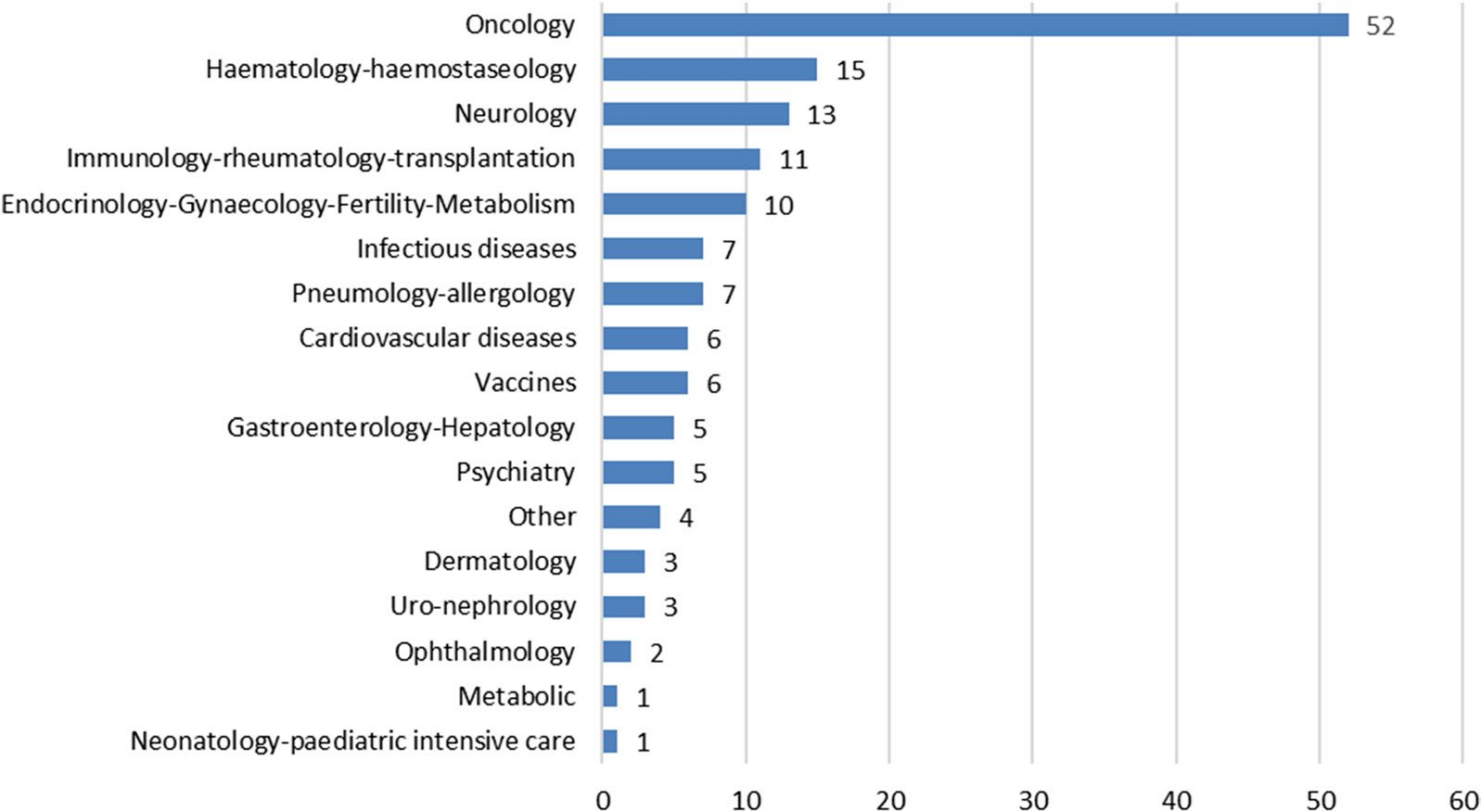
Common requests by therapeutic area for FDA breakthrough and EMA PRIME designation 2016–2020

Figure 1 shows common requests by therapeutic area. The majority, 52 of the 151 (34%) pertained to oncology products, followed by 15 (10%) for hematology/hematoseology, and 13 (9%) in Neurology. These three therapeutic areas are also those with the most requests for PRIME and for BT.

Of the 151 requests submitted for both PRIME and BT, 49 (32%) were submitted within 60 days of each other and 71 (47%) within 120 days. This pattern was consistent from year to year over the period of our cohort.

As shown in Table 2, the agencies reached concordant outcomes to grant or deny access to the respective program in 93 of the 151 (61.6%) common requests: 42 were designated as both BT and PRIME, 51 were denied both designations (or withdrawn by the sponsor). The agencies reached different outcomes on designation in 58 cases (38.4%). Table 2 also provides an overview comparison of the outcomes in oncology (the most common therapeutic area) vs non-oncology indications, which shows similar proportions of concordant and divergent outcomes.

**Table 2:** Comparison of EMA and FDA outcomes on common requests for PRIME and BT, overall and oncology products

EMA and FDA outcomes on PRIME/BT request	All common requests (*n* = 151)	Common requests oncology (*n* = 52)	Common requests non-oncology (*n* = 99)
Concordant	93 (61.6%)	30 (57.7%)	63 (63.6%)
Granted by both	42	14	28
Denied^a^ by both	51	16	35
Divergent	58 (38.4%)	22 (42.3%)	36 (36.4%)

^a^Requests withdrawn by the sponsor prior to finalization of the outcome were accounted as ‘denied’

### Comparison of BT Plus RMAT and PRIME Agency Outcomes

When also including RMAT products in our comparison the number of common product submissions to both Agencies increased to 176, and concordance in designation determinations remained similar (63.6%), as shown in Table 3.

**Table 3:** Comparison of EMA and FDA outcomes on common requests for PRIME and BT plus RMAT, overall

EMA and FDA decisions on PRIME/BT + RMAT	All common requests (*n* = 176)
Concordant	112 (63.6%)
Granted by both	55
Denied^a^ by both	57
Divergent	64 (36.4%)

^a^Requests withdrawn by the sponsor prior to finalization of the outcome were accounted as ‘denied’

### Reasons for Different (Divergent) Outcomes: BT and PRIME

For the 58 products with different outcomes, reasons underlying the divergence in FDA and EMA conclusions on PRIME and BT are shown in Table 4. For all 58 products we reviewed internal agency records to ascertain whether the proposals to both Agencies were based on the same data. We identified only 7 cases where data submitted were different. Of the remaining 51 cases, some reasons for divergent outcomes emerged. In four cases, different available treatment alternatives across regions which affected the assessment of whether the subject product may bring a major therapeutic advantage/significant improvement over existing therapies. In 13 cases, a difference in program implementation policy was the key reason for divergence. For example, among these 13 cases, 10 were denied by EMA because of its policy not to designate products too far advanced in development other than in exceptional circumstances. The majority of these ten cases occurred within the first 2 years after PRIME launched. For the 34 remaining products where the same data were submitted but the Agencies concluded differently, we could not identify a reason for the divergence other than differences of interpretation of the same data.

**Table 4:** Reasons for divergent outcomes on common applications for breakthrough (FDA) and PRIME (EMA)

Reason for divergence	*N* (total = 58)
Different data submitted	7
Differences of program implementation policy	13
Differences of currently available treatment across regions	4
Differences in interpretation of same data	34

### Reasons for Different (Divergent) Outcomes: BT Plus RMAT and PRIME

For the 64 products with different outcomes the main reasons underlying the divergence in FDA and EMA on PRIME and Breakthrough are shown in Table 5. The reasons for divergence were similarly distributed when adding RMAT.

**Table 5:** Reasons for divergent outcomes on common applications for breakthrough plus RMAT (FDA) and PRIME (EMA)

Reason for divergence	*N* (total = 64)
Different data submitted	9
Differences of program implementation policy	13
Differences of currently available treatment across regions	4
Differences in interpretation of same data	38

## Discussion

When we began tracking submissions to EMA’s PRIME and FDA’s BT in 2016, our expectation was that there would be many applications in common, as both programs focus on facilitating development of medicines addressing important unmet medical needs, which are rarely limited to one geographic area. Thus, we were surprised to find that most BT and PRIME eligibility requests from 2016 to 2020 were not common to both agencies: 43% of PRIME submissions had corresponding submissions for BT, and only 21% of BT submissions had corresponding PRIME submissions. The lower percentage of BT submissions with a corresponding PRIME submission may be attributable, especially in the earliest part of our cohort, to the newness and lack of industry familiarity with the program. It can be partially explained by the broader scope of eligibility for FDA programs. For example, FDA BT has no limitations on the number of separate designations for new indications of already marketed products nor how late in development a sponsor can submit a request. EMA, on the other hand, has sought to direct PRIME resources to products early in development. Other possible factors are many, such as the significant number of SME sponsors which may work primarily in one of the two jurisdictions, sponsors’ resource limitations, their intended market strategy, and/or their familiarity with FDA and EMA programs. Our data do not allow us to ascertain whether any of these are operative.

For cases where sponsors submitted requests to both Agencies, it is interesting to note the timing of submission. About a third were submitted very close in time (within 60 days) and about half within 120 days. It may speak to the fact that companies are or became familiar with both programs over the period of the cohort’s operation. We were expecting common requests to be submitted more closely in time as we examined our cohort year by year, but we did not observe a clear pattern toward this.

For the 151 products submitted for both BT and PRIME in the current cohort, concordance in outcomes to grant or deny designation was almost two thirds (62%), suggesting generally similar perspectives across international experts on the potential value of the products under study. Our numbers are too small to draw conclusions about whether the two thirds rate of concordance, overall, is similar across therapeutic areas, but it held for oncology products and pooled, non-oncology products. The patterns and findings did not change when we added RMAT to our comparisons.

Previous work has focused on the growth of scientific and technical collaborations of FDA and EMA over more than a decade along with increasing concordance in decisions about whether to approve marketing authorization applications [6]. As such, one might expect that decisions on data that form the basis for expedited development advice designations might follow a similar path. However, an important factor with BT/RMAT and PRIME is the often limited and preliminary nature of the evidence used to support designation, calling for more judgement on potential for translation to clinical benefit.

We sought to understand reasons for divergent designation outcomes across the two Agencies. Our first question was whether the same data were submitted to both. With access to both Agencies’ internal databases, for BT and PRIME we could only identify 7 of 58 divergent cases in which the applications were supported by different data. For these 7 products with newly generated data, the BT and PRIME requests were submitted on average 16 months apart (vs 5 months apart for those submitted with the same data). The remaining 51 cases fell under the other two possible reasons for divergent outcomes: program implementation policy reasons and differing availabilities of alternative treatment options across regions.

For the 34 remaining products where the same data were submitted but the Agencies concluded differently, we were unsuccessful at finding patterns characterizing their divergence. EMA denied 23 of the 34 requests while FDA denied 11 of 34. The 34 products spanned many therapeutic categories but, as was the case for in common applications, the only category with enough products on which to comment was oncology (7/34). EMA denied all seven and FDA accepted all seven, and all seemed to rest on the two agencies reaching different conclusions on sufficiency of evidence to warrant designation. For some among the seven cases, the divergence was related to quantity of data, in others strength of the findings in early trials, and in still others whether the endpoint measured in early trials was known to be clinically meaningful in the subject condition.

We did not seek to assess consistency of judgements within Agencies, as the products and diseases were so varied. In the end, we could only conclude, based on our limited cohort of 5 years, that decisions on BT, PRIME (and RMAT) designation are a matter of judgement on how preliminary data may predict efficacy or result in clinically meaningful benefit over existing therapy. For products early in development, experts reaching different conclusions about the significance of preliminary data and what it portends is expected.

Our analysis has provided a comparison of sponsor proposals and regulatory decisions, but our greater interest is in opportunities for synergistic efforts to expedite development of potentially important products destined for patients worldwide. Thus, an obvious question is what happens once a product is designated as BT or RMAT and PRIME: do FDA and EMA share their views with each other or otherwise collaborate? Though it is not a stated goal of either program, such collaboration on a designated product’s global development could lead to important benefits for patients and public health.

FDA and EMA have a number of forums to collaborate on challenging aspects of regulatory science, such as are often presented by products designated PRIME, BT, or RMAT. These collaborations range from standing technical working groups (often called “clusters”) [7] to Parallel Scientific Advice (PSA) [8]. Products with BT, RMAT, and/or PRIME designations may be discussed on an ad hoc basis in an individual cluster when proposed by one or the other Agency. It is important to note that the clusters are regulators-only forums and not intended to address requests by industry. A more detailed examination of the Agencies’ records for agendas of clusters over the years of our cohort could shed more light on how these informal mechanisms intersect these programs to expedite development.

Currently, the only formal mechanism for FDA and EMA to advise a sponsor collaboratively is PSA. This program, in place since 2009, allows a sponsor to specifically request advice from EMA and FDA on critical product development plans at the same time. Under PSA, the same submission is provided to both Agencies, who each conduct their own assessment, following which they discuss their assessments bilaterally before meeting together with the company. Each Agency provides its own formal advice letter per usual procedure. While not a goal of PSA, through the process it is common that the two agencies’ experts find their perspectives and advice in alignment. When they do not, they are able to help sponsors consider how to address the different advice efficiently going forward. We were able to determine that during the period of the cohort here, only one of the 42 products granted both BT and PRIME (55 if RMAT is included) was the subject of formal Parallel Scientific Advice. Yet, interestingly, in a survey of industry conducted by EMA as part of their 5-year review of the experience with PRIME, there were comments made about the desirability of prioritization of PRIME products for EMA and FDA scientific advice interactions [9].

## Conclusion

Working toward better engagement on global development strategies is in the best interests of patients and public health. Products designated as BT/RMAT and PRIME are those for which both EMA and FDA have found the potential to address an important unmet medical need, in other words of potential public health value and for which efficient development is highly desirable. With this in mind, our wish is that Agencies and sponsors will continue to take advantage of existing collaborative opportunities, such as parallel scientific advice, and work to identify fresh approaches to support global development of products for unmet medical needs.

## Disclaimer

The views expressed in this article are the personal views of the author(s) and may not be understood or quoted as being made on behalf of or reflecting the position of the regulatory agency/agencies or organizations with which the author(s) is/are employed/affiliated.

## Footnotes

1 We use the term “sponsor” to represent applicants for PRIME, BT, or RMAT; most often a pharmaceutical company.

2 Requests withdrawn by the sponsor prior to finalization of the outcome were accounted as ‘denied’. If a sponsor resubmitted requests to both Agencies after being initially denied, the comparison was made for the initial requests and for the resubmitted requests.
